# Revisiting the role of pathogen diversity and microbial interactions in honeybee susceptibility and treatment of *Melissococcus plutonius* infection

**DOI:** 10.3389/fvets.2024.1495010

**Published:** 2024-12-19

**Authors:** Elizabeth Mallory, Gwendolyn Freeze, Brendan A. Daisley, Emma Allen-Vercoe

**Affiliations:** Department of Molecular and Cellular Biology, University of Guelph, Guelph, ON, Canada

**Keywords:** European foulbrood, *Melissococcus plutonius*, clonal complex, secondary invaders, microbial ecological interactions, probiotics

## Abstract

European Foulbrood (EFB) is a severe bacterial disease affecting honeybees, primarily caused by the Gram-positive bacterium *Melissococcus plutonius*. Although the presence of *M. plutonius* is associated with EFB, it does not consistently predict the manifestation of symptoms, and the role of ‘secondary invaders’ in the disease’s development remains a subject of ongoing debate. This review provides an updated synthesis of the microbial ecological factors that influence the expression of EFB symptoms, which have often been overlooked in previous research. In addition, this review examines the potential negative health consequences of prolonged antibiotic use in bee colonies for treating EFB, and proposes innovative and sustainable alternatives. These include the development of probiotics and targeted microbiota management techniques, aiming to enhance the overall resilience of bee populations to this debilitating disease.

## 1 Introduction

The pollination services of honeybees contributes to revenue of over USD $18 billion in crop production annually in the United States and more than 80% of all crops depend, to varying extents, on insect pollination (1, 2). Overall global honeybee numbers have grown since 1961, yet in that same time period, the area of insect pollinated crops has grown by more than 300%, highlighting the increased demand for such pollinators (2, 3). Over the past few decades, beekeepers from many nations around the world (including, for example, EU, Mexico, Canada, New Zealand and Iran) have experienced an alarming and continually increasing rate of honeybee mortality (4–6). In 2022–2023, total Canadian honeybee colony loss was estimated at 48%, over three times the approximate sustainable limit (5). Major colony losses have been attributed to a combinatorial effect of pesticide exposure, natural habitat loss, nutritional deficiencies, pathogen infection, and more (7). Due to the importance of honeybees to the environment and the agricultural system, researchers and industrialists seek strategies to reduce and treat the stressors challenging honeybees.

One stressor, European Foulbrood (EFB), is a major honeybee disease which affects larvae of managed Western and Asian honeybees (8). Despite the serious consequences of infection (larval mortality and subsequent colony population decline) this disease is poorly understood.

EFB is caused by the pathogenic bacterium, *Melissococcus plutonius*, where larvae become infected through oral ingestion via dietary contamination by asymptomatic worker bees. The pathogen then colonizes the larval gut, outcompeting the host for nutritional substrates, ultimately leading to host starvation. New evidence suggests *M. plutonius* crosses the host gut epithelium layer to cause septic infection (9). Infected larvae undergo a distinctive transformation, transitioning in color from a white to a yellow hue and manifesting a deflated appearance before succumbing to the infection. A colony affected by EFB experiences sporadic and irregular brood development, leading to diminished honey output, reduced pollination efficiency, and, in severe cases, complete colony collapse. For a beekeeper, the direct monetary impact of a single infected colony exhibiting mild disease is estimated to be ~ USD $215 in treatment and equipment replacement costs (10).

Due to its potential economic impact, and the ease of spread of the disease between hives, EFB is categorized as a reportable disease in many countries, as listed in the World Organization for Animal Health (WOAH). In terms of treatment for infected hives, beekeepers have rather limited options. Oxytetracycline, a broad-spectrum antibiotic that interferes with bacterial protein synthesis, has been used for years in many countries as both a treatment for, and a prophylactic against, infection. However, this antibiotic is associated with high rates of EFB recurrence (27%) and honeybee larval toxicity (11–13). In addition, despite strict adherence to current treatment guidelines by beekeepers, honeybee colonies continue to be commonly infected by *M. plutonius*, demonstrating an acute need for research to elucidate strategies for effective EFB treatment and eradication.

## 2 History, cultivation challenges, and pathogen diversity

Although *M. plutonius* was identified as the causative agent of EFB over 100 years ago, researchers have faced significant challenges in studying this pathogenic bacterium because of its fastidious growth requirements. Most *M. plutonius* strains require strict anaerobic conditions for growth as well as a culture medium containing specific nutrients (14). Although recent advances have been made with regard to improved isolation methods and media types, many strains of *M. plutonius* are still difficult to cultivate and tend to grow very poorly under laboratory conditions, thereby hindering research.

Further complicating matters is the fact that *M. plutonius* can often be detected in asymptomatic hives, suggesting that it is a common pathobiont of honeybees, i.e., its presence may not necessarily cause apparent signs of disease. It is been long speculated that the pathobiont behavior of *M. plutonius* is a result of distinct genetic determinants impacting virulence (15). Consistent with this, Arai et al. performed comparative characterization of 33 *M. plutonius* field isolates and identified two clear phenotypic groupings, denoted as ‘typical’ or ‘atypical’ based on their biochemical and culture characteristics relative to the features of the type strain, *M. plutonius* ATCC 35311 (Table 1). Using *in vitro* in larvae infection assays, it was found that atypical isolates caused higher mortality rates compared to typical isolates; specifically, atypical isolates killed between 70–90% of infected larvae within 5 days, whereas typical isolates killed less than 20% even when given the same infectious dose (16). From the limited number of studies that have been done, it can be surmised that typical strains have a wide variance in their infectious capabilities, whereas atypical isolates consistently cause high mortality (17).

**Table 1:** Phenotypic differences between typical and atypical *Melissococcus plutonius* as described in Arai et al. (2012) and Takamatsu (2023).

	Typical	Atypical
Clonal Complex(es)	CC3, CC13	CC12
Oxygen Requirements	Strict anaerobe	Facultative anaerobe
Potassium Supplementation for Growth^a^	Required (K > Na)	Optional
Carbohydrate Utilization	Limited carbohydrate utilization profile	Wider carbohydrate utilization profile
Regions and Countries Prevalent	Widespread through all continents (except Antarctica)	Japan, Mexico, Canada, United Kingdom, Switzerland, USA
*In Vitro* Lethality	Low to high mortality (dependent on strain)	High mortality

a Potassium supplementation in growth medium to achieve a K:Na ratio, where K > Na.

Genomic investigations have shown that *M. plutonius* strains can be grouped into three ‘clonal’ complexes of *M. plutonius* (CC3, CC12, and CC13; Table 1). CC12 strains are of particular interest due to their virulence and prevalence in certain regions, and all display ‘atypical’ characteristics (18). CC3 and CC13 strains, seen as more predominant, display the ‘typical’ phenotypic characteristics, such as required low sodium and high potassium growth conditions. A summation of this can be found in Table 1.

## 3 Geographic distribution of EFB and detection of antibiotic resistance

Some of the oldest and most well-documented reports of EFB come from Canada in the early 20th century. In 1916, the “Apiary Inspection in Ontario” indicated that out of 5,367 colonies inspected, a total of 1,387 colonies (~25%) displayed symptoms of EFB (19). Following the introduction of oxytetracycline in the 1940s, EFB either disappeared or dropped to undetectable levels in many regions, and when disease outbreaks did occur, they were generally considered self-limiting with clinical symptoms often found to resolve spontaneously (20, 21).

In the past several years, there has been a re-emergence of severe EFB outbreaks in several regions of the world including the US, Canada, and Switzerland (22–24). The extent of this re-emergence, however, is likely underestimated, since EFB symptoms can be ambiguous and the disease itself is reportable in many countries, as per its assignment in WOAH. In Canada, the increase in symptomatic EFB infections was first observed in 2019 in colonies used for commercial blueberry pollination (23, 25). The phenomenon was initially thought to be related to immune deficits of bees feeding on nutrient-poor blueberry pollen. However, colonies not necessarily exposed to blueberry crops have also been affected, for example, a three-year molecular survey conducted in Alberta recently detected EFB in over 30% of the province’s colonies (10).

Insight regarding the prevalence of typical and atypical *M. plutonius* within hives can be gleaned from two studies using genetic surveillance. Arai et al. conducted random sampling of honeybee colonies across Japan and found that of infected colonies, 40% contained only typical *M. plutonius* strains, 6% contained only atypical *M. plutonius* strains and 54% were co-infected with both typical and atypical strains (26). De León-Door et al. confirmed similar findings (27). The higher proportion of co-infected hives suggests that atypical *M. plutonius* strains may gain an advantage when co-infecting with typical strains. Diverse, thorough sampling in regionally distinct areas is required to confirm this phenomenon. Additionally, further research is needed to elucidate why typical/atypical strain co-infections may be beneficial for disease progression.

Compared to other areas of the world (e.g., the United States Japan, Switzerland, and United Kingdom) (17, 28) there has been limited genetic investigation of the EFB strains present in Canada or their proportionality. One exception is the recent study by Thebeau et al., which assessed pathogenicity and determined clonal complex genotypes of 4 clinical *M. plutonius* isolates derived from EFB outbreaks in Saskatchewan, Alberta, and British Columbia (29). Notably, 75% of the *M. plutonius* isolates found were identified as CC12 strains that were highly pathogenic (associated with ~58–70% mortality of larvae) based on *in vitro* larval infection assays (note that the convention for the use of ‘*in vitro* larval’ in this field tends to refer to studies of larvae within a lab environment).

One Canadian CC12 isolate, 2019 BC1, has displayed a minimum inhibitory concentration (MIC) of 16 μg/mL to oxytetracycline (30). This MIC is higher than the concentration used for standard EFB treatment, and therefore could constitute this strain as oxytetracycline resistant. In a later study by Takamatsu et al., 77 *M. plutonius* isolates from Japan were examined, revealing an additional four strains [comprising three CC3 and one CC12 strain(s)] resistant to oxytetracycline, based on MICs of 16 μg/mL (31). The emergence of oxytetracycline resistance in *M. plutonius* appears independent of clonal complex type, and dates to at least 2008 when the Japanese isolates were first isolated. Elucidation of the mechanism of resistance to oxytetracycline in *M. plutonius* is warranted to obtain a comprehensive understanding of its prevalence and risk and to explore potential avenues for intervention. Genome level analysis of *M. plutonius* isolates from EFB-infected hives sampled globally are needed in order to better understand the relationship between genetic diversity and virulence in this pathogen.

## 4 Virulence determinants of *Melissococcus plutonius*

### 4.1 Molecular determinants

As described above, *M. plutonius* isolates can be grouped into three clonal complexes with distinct phenotypic and genomic characteristics. Nonetheless, data collected from over 500 isolates across 18 countries show a lack of consensus with regard to pathogenicity of *M. plutonius* based solely on clonal complex genotype (32). These inconsistencies may be related to the acquisition by some strains of a virulence plasmid (pMP19) which encodes a putative ETX/MTX2 toxin family protein with insecticidal properties, provisionally named ‘melissotoxin A’. For example, pMP19 positive CC3 strains produce mortality rates of >91% during larval infection assays (32). Demonstrating a causal link, Nakamura et al. showed that loss of the pMP19 plasmid abrogated pathogenicity and that larvae infected with pMP19-cured CC3 strains experienced only a 6% mortality rate (33). In the same study, pMP19 failed to explain the virulence of CC12, with both pMP19-positive and -negative strains resulting in up to 90–100% larval mortality rates (33). Given this extreme virulence combined with a lack of understanding of the underlying mechanisms, ‘atypical’ CC12 *M. plutonius* strains are considered the most dangerous strains which pose the greatest risk for severe EFB outbreaks.

Other genetic determinants have been proposed to play a role in virulence, such as the presence of the gene encoding tyrosine decarboxylase, a catalytic enzyme responsible for the conversion of tyrosine to tyramine and carbon dioxide (34). A buildup of tyramine has been linked to development of classic EFB symptoms in larvae (35). The tyrosine decarboxylase gene is present and functional in strains belonging to CC12 and CC3 only, and presents a compelling potential virulence determinant of these clonal complexes compared to CC13 (16, 36).

### 4.2 Interplay between ‘secondary invaders’ and EFB disease onset

**Figure 1:**
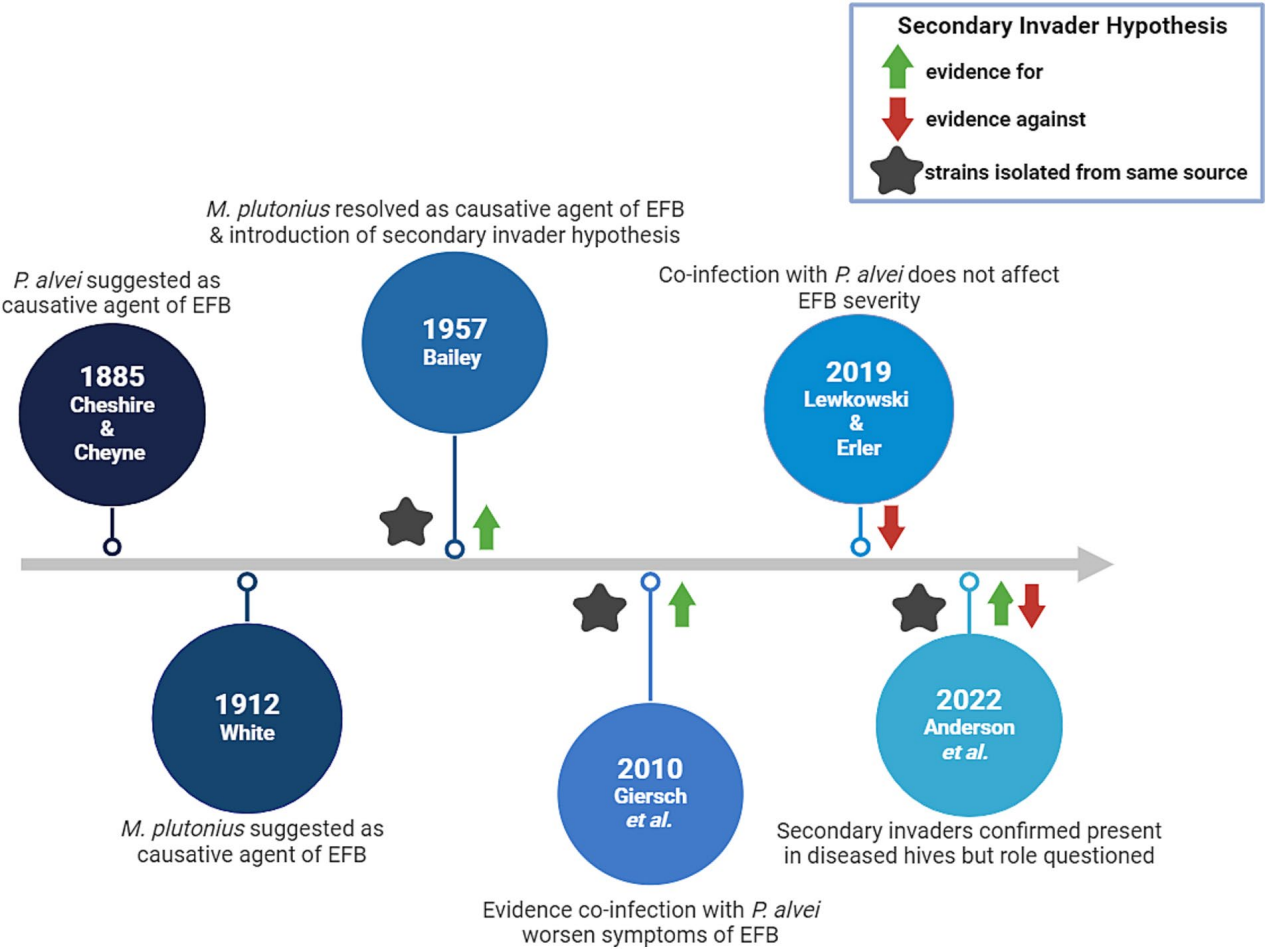
Timeline of major findings indicating the causative agents of EFB.

EFB disease severity may be influenced by larval co-infection with so-called ‘secondary invaders’, i.e., other microbial species that can work in tandem with *M. plutonius* to cause symptoms. Several studies have focused on this phenomenon and the major findings can be found in Figure 1.

Originally, *Bacillus alvei* (since reclassified as *Paenibacillus alvei*) was indicated as the causal organism responsible for producing EFB, since this bacterial species was found abundantly in larvae with EFB symptoms (37). This finding was called into question by White, who proposed the causal organism of EFB as *M. plutonius* (38). More than four decades later, Bailey failed to reproduce EFB symptoms in larvae when using only pure cultures of *M. plutonius* (39), but later was the first to suggest that *M. plutonius* may not act alone to cause disease; *M. plutonius* was identified as the primary etiological agent of EFB in addition to a supplementary role of several other bacterial species, including *Enterococcus faecalis*, ‘*Achromobacter eurydice’*, or *Paenibacillus alvei* (40). The source of these accomplice strains is not always clear, but several reports have suggested that *Paenibacillus* and *Enterococcus* spp. can be vectored by ectoparasites such as Varroa mites and small hive beetles (41–44). However, the roles and impact of *E. faecalis*, *A. eurydice* and *P. alvei* on EFB disease progression have been contentious, with contradictory evidence of their involvement.

Not discussed in detail in this review, confusion surrounding the actual identity of the proposed secondary invader first described as ‘*A. eurydice*’ has been called into question, thus rendering past research regarding its role in EFB progression contentious. This controversy is discussed in depth in Erler et al. (45).

Testing the secondary invader hypothesis *in vitro* in larvae, Giersch et al. reported that the specific combination of *M. plutonius* and *P. alvei* field isolates derived from the same EFB infected colony could reliably produce symptoms characteristic of EFB (46). Furthermore, this combination increased mortality in lab-reared larvae when compared to monoculture infection with only *M. plutonius* (46). Conversely, Lewkowski and Erler found no effect of experimental co-infection when using strains of *M. plutonius* 49.3 and *P. alvei* LMG 13253 in larvae *in vitro* (47). Anderson et al. confirmed that both *P. alvei* and *E. faecalis* were present in EFB diseased colonies, but did not necessarily find that the presence of one or both species correlated with disease progression (41). *In vitro* in larvae it has been shown that *E. faecalis* co-infection with *M. plutonius* did not result in more severe infection compared to each species alone, however this may have been because the strains used for this work were not isolated from a diseased colony (47). Analysis of the role of ‘*A. eurydice’* in EFB, has not been further investigated, to the best of our knowledge.

Although there has not been much work done to understand synergistic infections with multiple microbes in cases of EFB disease, the work that has been done offers some compelling evidence that in addition to *M. plutonius*, other microbes are involved. Whether microbial adaptation is required of *M. plutonius* in order to infect with a given secondary invader, or whether existing strain-level differences influence the likelihood of disease is undetermined.

The combined effects of primary and secondary pathogens, along with unknowns about clonal complex (CC3/CC12/CC13), create a complex web of interactions driving the probability of EFB disease incidence and severity that is yet to be fully deciphered. Unraveling this complexity will require comprehensive research but is necessary in order to devise effective EFB management strategies and ensure honeybee health and sustainability.

## 5 Use of honeybee symbionts in the development of alternative EFB treatments

### 5.1 Natural defense mechanisms of the honeybee gut microbiota

The adult honeybee gut microbiota is highly conserved and plays a pivotal role in nutrient absorption, detoxification, and immune system modulation (48). Although it also provides host benefits, the gut microbiota of honeybee larvae is more variable than that of adults, showing lower abundance of microbial species, and often the presence of environmental microbes (41). In terms of the role of the honeybee microbiota in EFB interactions, only a limited number of studies have been completed to date.

One study assessed the microbiomes of two types of adult worker bees derived from apiaries with clinical signs of EFB infection: a symptomatic type and an asymptomatic type (49). Unsurprisingly, there were large differences in the abundance of *M. plutonius* among the bees, with symptomatic bees harboring a 75-fold higher load of *M. plutonius* compared to asymptomatic bees (49). However, significant differences in the abundance of other microbial taxa among the two types were also identified (49). Symptomatic bees not only possessed a higher incidence of *M. plutonius*, but also higher proportions of *Fructobacillus fructosus*, *Apilactobacillus kunkeei*, *Gilliamella apicola*, *Frischella perrara*, and *Bifidobacterium indicum* (49). Species of *Snodgrassella alvi*, *Lactobacillus melliventris, L. helsingborgensis* and *L. kullabergensis*, the latter 3 of which are lactic acid-producing bacteria (LAB), were found in higher abundance in worker bees from asymptomatic colonies compared to their counterparts and it is therefore possible that their presence plays a role in EFB disease protection or suppression, especially early on in development (49). It is notable that in a further study where microbiomes of larva were screened, LAB were also negatively associated with *M. plutonius* (50). In addition, the lack of a single acetic acid-producing bacterium (*Bombella apis*) was found to be uniquely associated with atypical EFB symptomatology (50).

Due to the correlative nature of typical microbiome research, results indicating the positive or negative correlation of a certain species with *M. plutonius* load is a cause-and-effect dilemma. One member being in higher abundance when *M. plutonius* levels are higher could indicate that this species is spurring growth of *M. plutonius*, or, the growth of that member could be a response to independent *M. plutonius* growth. As such, mechanistic microbiome research must be carried out to determine the nature of this correlation, through methods such as infection assays with individual gain or loss of specific microbiome members or crosswise microbiota transplants.

Overall, these results raise questions about whether symbiotic bacteria may play a central role in the promotion of colony health in the presence of *M. plutonius,* mitigating the risk of EFB development. While our understanding of bee microbiota ecology and interactions in EFB is still nascent, these findings suggest that certain beneficial bacteria may impede the colonization of *M. plutonius* and act as a natural barrier against infection. As such, the potential for harnessing certain beneficial microbes as prophylactics for EFB disease prevention is of interest.

### 5.2 Probiotics as a promising therapeutic alternative

The development of beneficial microbes as probiotics for use in hive management is a relatively new and expanding strategy for apiarists. Several groups have successfully identified honeybee-derived strains of LAB representative of *Lactobacillus*, *Apilactobacillus,* and *Pediococcus* spp. which were able to strongly inhibit *M. plutonius* growth *in vitro* (51–53). The mechanism(s) of this inhibition remains unexplored, however some LAB produce metabolites, such as organic acids, bacteriocins (a class of small antimicrobial peptides), or bacteriocin-like inhibitory substances (BLIS) that are known to inhibit pathogen growth. For example, it has been found that the cell-free supernatant of an isolate of *Apilactobacillus kunkeei* (formerly *Lactobacillus kunkeei*), thought to be one of the most abundant LAB species in honeybees, has growth inhibitory effects against *M. plutonius* (54). This finding was later confirmed by Zendo et al., who also demonstrated that the mechanism of *M. plutonius* growth inhibition was through the production of a bacteriocin, kunkecin A, by *A. kunkeei* (52). Additionally, a recent study by Leska et al. revealed that 55 out of 103 tested LAB strains showed antagonistic activity against *M. plutonius*, with one strain of *Ligilactobacillus salivarius* demonstrating the most notable growth inhibition among them (51). As well, the cell-free supernatant of *L. salivarius*, as well as strains of *Pediococcus parvulus* and *Levilactobacillus brevis* also notably inhibited *M. plutonius* growth (51). Mojgani et al. also found that the cell-free supernatants from LAB species *Lactobacillus acidophilus*, *Lacticaseibacillus rhamnosus*, *Lactiplantibacillus plantarum*, *Lactobacillus apis* and *Pediococcus acidilactici* potently inhibited *M. plutonius* growth, with the production of organic acids, bacteriocins or BLIS being proposed as critical mediators of this activity (53).

These results support microbiota associations and suggest that certain LAB may be prime probiotic candidates. However, each of these *in vitro* studies used a single *M. plutonius* strain as their test organism: the type strain ATCC 35311. Since (as described above) atypical *M. plutonius* strains have divergent virulence factors and culture requirements, the inhibitory activity seen with the type strain may not be generalizable to all EFB etiologic agents.

That said, two studies have screened honeybee-derived bacterial strains for antagonistic activity toward atypical *M. plutonius* strains. Wu et al. found that one *Lactobacillus* sp. strain (Acaj3) exhibited strong inhibitory activity against atypical *M. plutonius* (strain DAT 561) *in vitro* (55). In direct comparison with antibiotics, the same authors found that Acja3 was comparable to tetracycline in its antibacterial activity against *M. plutonius* and could reduce *in vitro* larval mortality by ~50% during infection assays. A notable advantage of some beneficial bacteria is their bactericidal properties (i.e., killing capacity) in comparison to tetracycline, which exerts bacteriostatic effects (i.e., growth inhibitory without killing) (56). Various *Bifidobacterium* spp. have also been investigated for their antagonism against two atypical *M. plutonius* strains (DAT 561 and DAT 351), and some were found to exhibit antagonistic effects against its growth (57).

To date, in total, only three *M. plutonius* strains (ATCC 35311, DAT 561, and DAT 351) have been tested for their direct inhibition by potential probiotic strains *in vitro*. As it is becoming clear that different *M. plutonius* strains can possess a multitude of different virulence factors and culture requirements, it is also becoming clear that there is a gap in knowledge about the likely applicability of these probiotic strains to protect against EFB disease in general. Furthermore, while work in the field shows intriguing results, future studies will be imperative to understand the effect of these candidate strains *in vivo*, especially to account for the impact of other gut microbial members and host-factors on probiotic efficacy.

In support of this, in the field, a recent study in Italy (979 colonies/22 apiaries) showed that *Lactiplantibacillus plantarum* LMG P-21806 treatment reduced EFB prevalence from 4.5 to 2.5% over a 6 month period *in vivo* (58). Another *in vivo* study in California (33 colonies/2 apiaries) indicated a three-strain lactobacilli consortium (LX3; composed of *Lactiplantibacillus plantarum* Lp39, *Lactobacillus rhamnosus* GR-1 and *Apilactobacillus kunkeei* BR-1) could reduce *M. plutonius* burden by five-fold over a 20 week period (59). This work is an especially promising start to understanding the role of probiotic treatment *in vivo*; however, future studies are required to better characterize these interactions and outcomes on EFB. Furthermore, since these studies utilized natural EFB infections, no genotype or *M. plutonius* strain information was provided, which is an area in need of further characterization.

While the potential effectiveness of probiotic strains/treatments have been described for mitigating *M. plutonius* growth and honeybee disease, it should be noted that not all probiotics show equally promising results. In recent years, commercial bee probiotics (e.g., SuperDFM®-HoneyBee™, the most popular honeybee probiotic in the United States) have been found unable to colonize or restore/antibiotic rescue the honeybee gut microbiome (60–62).

A strong hypothesis regarding this discrepancy is due to the composition of probiotics, as commercial products are typically comprised of non-native microbiota that have a slim chance of establishment and subsequent survival in the honeybee gut (60–62). The efficacy of probiotics composed of a mixture of native bee microbiota, however, have been shown to colonize the honeybee gut with success and are a promising area of future work, which could potentially be applied in the treatment of EFB (62, 63). That said, it is important to consider that further *in vivo* work is imperative before drawing conclusions regarding the therapeutic use of probiotic strains for disease management in honeybees.

Additionally, the influence of geographic variation and environmental factors is crucial to the interpretation of research outcomes, and there is not enough data to evaluate this at present. This limitation leaves substantial gaps in our understanding of how probiotics interact with *M. plutonius* strains, both typical and atypical. Understanding these dynamics, and others, is essential for developing effective treatment strategies that can be adapted to diverse environmental conditions and regional characteristics. A summary of candidate species and tested strains discussed in this section are found in Table 2.

**Table 2:** Candidate probiotics demonstrating strain-level *in vitro* or *in vivo* activity against select strains of *Melissococcus plutonius*.

Strain(s) causing antagonism	*M. plutonius* strain(s) tested against	Basic methodology	Reference
*Ligilactobacillus salivarius* 9AN	ATCC 35311 (typical)	*in vitro* inhibition assay	(51)
*Apilactobacillus kunkeei* FF30-6	ATCC 35311 (typical)	*in vitro* inhibition assay	(52)
*Lactobacillus acidophilus* ZN06 *Lactobacillus apis* ZN027 *Lacticaseibacillus rhamnosus* ZN012 *Lactiplantibacillus plantarum* ZN098 and ZN3b-ab *Pediococcus acidilactici* ZN016	ATCC 35311 (typical)	*in vitro* inhibition assay	(53)
*Lactobacillus apis* sp. nov. strain R4B^T^	ATCC 35311 (typical)	*in vitro* inhibition assay	(78)
*Lactiplantibacillus plantarum* strains: LP 31, LP 42, LP 148 and LP 179 *Apilactobacillus kunkeei* strains: ALK 181, ALK 222, ALK 268 and ALK 385	ATCC 35311 (typical)	*in vitro* inhibition assay	(79)
*Bifidobacterium* sp.: Acj BF1-Acj BF11	DAT561 (atypical) DAT351 (atypical)	*in vitro* inhibition assay	(57)
*Lactobacillus* sp. Acja3	DAT561 (atypical)	*in vitro* inhibition assay *in vitro* larval assay	(55)
*Lactiplantibacillus plantarum* LMG P-21806	undetermined (natural infections, presumedly typical)	*in vivo* (field-level, observational)	(58)
LX3 Mixture: (*Lactiplantibacillus plantarum* Lp39, *Lactobacillus rhamnosus* GR-1 and *Apilactobacillus kunkeei* BR-1)	undetermined (natural infections, presumedly typical)	*in vivo* (field-level, observational)	(59)

### 5.3 Other therapeutic alternatives to counteract EFB disease

There are several approaches currently used to try to control the incidence of EFB disease. Hygienic breeding techniques can generate bees that efficiently detect and remove dead and infected larvae, which is expected to reduce EFB spread. Palacio et al. found that colonies selectively bred for hygienic behavior experience diminished rates of EFB, yet Fowler et al. has since suggested that there was no effect of hygienic bee behavior on the development of EFB (64, 65). These conflicting studies may be a result of the rate at which a given *M. plutonius* strain is able to kill larvae. For example, CC12 strains tend to kill larvae before capping, whereas some CC3 *M. plutonius* can cause mortality at a slower rate such that larvae are capped before death (66); all honeybees will dispose of dead larvae in uncapped cells, suggesting that hygienic behavior would not be an advantage in infections caused by CC12 *M. plutonius* (67).

Some honeybee lines also naturally resist EFB, hinting at a potential for advancements in bee breeding, however the host genetic determinants of resistance are currently unknown (47, 68). Additionally, natural substances such as essential oils show antimicrobial properties against several microbes and parasites that cause various bee diseases, but these substances require careful use to avoid toxicity in the bees themselves, and to prevent the development of microbial resistance (69). In this respect, tea tree oil application has been shown to inhibit *M. plutonius in vitro* and was determined to be non-toxic to honeybee larvae at effective levels (70).

A proposed factor in the development and progression of EFB is malnutrition. As previously stated, a suggested mechanism of disease involves host-pathogen competition for nutrients. During infection, malnutrition may also result from the immune system’s energy demands, which divert resources away from nutrient uptake and metabolism (71). Pollen, a critical component of royal jelly and a key dietary addition during later larval development, may significantly influence disease outcomes. Outside the context of infection, larvae fed different pollen sources during the pre-pupal stage exhibit varying survival rates (72). Additionally, pollen type has been shown to affect pre-pupal weight, while pollen dose impacts development speed (72). These findings underscore the importance of pollen type for healthy larval development, even under normal conditions.

This evidence supports the notion that supplementing honeybee colonies with pollen or pollen substitutes could improve overall health and potentially reduce disease susceptibility, especially in colonies with insufficient pollen sources or storage. Researchers have demonstrated that pollen supplementation reduces *Nosema* spp. infestations in adult bees; however, it also shortens the bees’ average lifespan (73). While this approach shows promise as a preventative measure, further research and refinement are needed to optimize its benefits. To our knowledge, no studies have directly examined the effects of pollen supplementation on *M. plutonius* infections.

Lastly, a treatment known as the ‘shook swarm’ method, involves transfer of EFB-infected adult bees to a new brood box supplemented with a sugar-oxytetracycline solution. This method results in decreased EFB symptoms and significantly reduces the reoccurrence rate compared to hygienic breeding, or the use of natural substances (11), although it runs the risk of contributing toward the development of antimicrobial resistance in bacteria associated with treated hives.

## 6 Areas requiring further research and future directions

### 6.1 Strain diversity and its role in disease progression

The escalating EFB crisis jeopardizes the honeybee population and, subsequently, their pollination services that contribute to agricultural efficiency. Partly attributed to the presence of atypical *M. plutonius* strains, EFB infections are becoming steadily more problematic and rising in occurrence. Although many atypical strains have been isolated from Japan, their presence has recently become apparent in other regions, including North America and Europe. Already determined to be phenotypically different in terms of growth requirements, the extent to which atypical strains differ in disease progression from their typical counterparts is still unclear. Continued genomic investigations of distinct strains *M. plutonius* strains, their associated virulence factors, and requirements for virulence expression need to be completed alongside confirmatory *in vitro* assays to clarify these discrepancies.

### 6.2 Defining the secondary invaders and their impact

Conflicting *in vitro* reports of the impact of secondary invaders could be explained by several different experimental factors which have been proposed to affect larval survival in other settings, such as: different larval rearing protocols (74), differing honeybee lineages with disparate innate resistance (68), or different experimental inoculation methods (75). To limit the impact of differing experimental design, future studies should look to incorporating a broader array of *M. plutonius* strains and using multiple larval rearing and larval inoculation protocols, akin to treatment groups. This would aid in determining whether virulence is indeed enhanced or remains unchanged in the presence of a variety of proposed secondary invaders, and not merely an effect of experimental design.

A further reason for the conflicting results so far seen between studies may be strain-specific co-adaptation within a given hive environment. For example, when *M. plutonius* and purported secondary invader strains were isolated from the same infected colony, synergism in EFB infection outcomes was seen; this was not repeated in a separate study using strains isolated from different sources (46, 47, 76). To determine this argument’s validity, a co-infection study should be conducted using *M. plutonius*-secondary invader pairings from unrelated and single isolation source(s). Furthermore, whether *M. plutonius* genotype also plays a role in interactions with secondary invaders remains in question. To the extent of our knowledge, no research to date has comprehensively compared the interactions of different *M. plutonius* clonal complexes with secondary invader partners. Studying the role of genotype in these interactions may also explain discrepancies seen in work to date, and is an area requiring future research.

These contradictory findings could also be a result of the misidentification of the secondary invader itself. Giersch et al. indicated *P. alvei* identity via Gram stain and morphological comparison of vegetative cells and endospores, yet several *Paenibacillus* spp. are highly similar in these characteristics. For example, the recently-described species *Paenibacillus melissococcoides,* also isolated from a honeybee colony experiencing EFB, is closely related to *P. alvei* as determined through 16S rRNA gene sequence comparison, and also forms endospores (77). Therefore, it is possible that the secondary invader which produced EFB symptoms and increased virulence when co-infected with *M. plutonius* in Giersch et al.’s study may be representative of another *Paenibacillus* spp. such as *P. melissococcoides.* Detailed molecular investigation regarding the true identity of strains used in these studies, would help to resolve species and strain co-occurrence in EFB-infected larva.

### 6.3 Probiotic efficacy to emerging strains

Discoveries regarding the determinants of EFB disease can be used to inform future alternative treatments. Current treatment options for EFB, such as oxytetracycline application, pose several unacceptable limitations, particularly in an era of increasing antimicrobial resistance, and with a dawning understanding of the importance of the honeybee microbiome in health. In contrast, probiotic approaches offer exciting new avenues, with many potential benefits. However, only limited work on the effectiveness of probiotics against EFB disease has been undertaken, and there remains a critical gap in understanding probiotic efficacy against divergent *M. plutonius* strains. Going forward, probiotic efficacy studies in the treatment and prevention of EFB should encompass multiple *M. plutonius* strains, including representatives of typical and atypical strains to demonstrate applicability in the field. Moreover, when considering prophylactic probiotic use in honeybees, developing researchers should aim to understand how these probiotics affect beneficial gut flora and as well as potential secondary invaders.
